# An Unusual Neurological Form of Acute Intussusception in an Infant: Late Diagnosis and Management

**DOI:** 10.1155/2019/5102615

**Published:** 2019-07-08

**Authors:** Ouidad Louachama, Noureddine Rada, Ghizlane Draiss, Karima Fouraiji, Mohamed Ouled Saiad, Mohamed Bouskraoui

**Affiliations:** ^1^Pediatric Department A, Mother and Child Hospital, Mohamed VI Marrakesh University Hospital, Marrakesh City, Morocco; ^2^Surgery Pediatric Department B, Mother and Child Hospital, Mohamed VI Marrakesh University Hospital, Marrakesh City, Morocco

## Abstract

Acute intussusception is one of the most common causes of intestinal obstruction in infants and small children and involves the invagination of one bowel segment into another. The clinical signs can be deceptively misleading when there is only one symptom or when an unusual symptom is in the foreground, especially in infants less than 1-year-old. We report a case of an infant with an acute intussusception where the neurological symptoms are predominant. The neurological form is the expression of major vascular narrowing of the collar of the intestinal intussusception; this form is rare and misleading and can be responsible for a delayed diagnosis.

## 1. Background

Acute intussusception in infants is a potentially serious obstructive disease. It is the invagination of one portion of the intestines into another and is the most common form of intestinal obstruction in infants. The vast majority of cases of intussusception is idiopathic and is thought to be due to hyperplasia of the lymphoid tissue in the Peyer's patches of the terminal ileum.

The classic form is typical, but there are atypical presentations, with systemic and/or neurological clinical signs in the foreground. If left untreated, it may lead to intestinal infarction, peritonitis, perforation, and death.

## 2. Case Report

A 6-month-old girl, the youngest of two siblings, vaccinated, with no particular pathological history, was admitted to the pediatric department for early postprandial vomiting three days ago, and at admission, she presented a generalized tonic-clonic convulsions and disorders of consciousness without refusal of feeding or excessive crying.

The physical examination revealed a temperature of 38°C, pulse of 126, and respiratory rate of 30, with moderate dehydration, a diuresis preserved and negative Multistix, and mucoïd stools. Her abdomen was soft, distended, not tender, and without masses. Her neurological examination showed a lethargic infant, not reactive and hypotonic. The anterior fontanel was open and soft, and the pupils were equal and reactive to light.

An abdominal ultrasound showed a severe meteorism impeding examination, and an abdominal radiograph did not reveal air fluid levels.

The investigation showed lymphocytic leukocytosis (leukocytes 15840/mm^3^; neutrophils 5290/mm^3^; lymphocytes 7520/mm^3^; hemoglobin 14.9 g/dl; hematocrit 44.6%; platelets 817,000/*μ*l) and functional renal failure (urea 0.95 g/l; creatinine 7.4 mg/l) without ionic disorders (sodium 145 mmol/l; potassium 4.4 mmol/l). Spinal tap, blood cultures, and stool cultures were sterile.

Three hours later, the patient presented a second seizure, bilateral miosis, worsening of consciousness disorder with a Glasgow score at 8/15, onset of bilious vomiting, and bloody stools.

She underwent an exploratory laparotomy for intussusception reduction and appendectomy, which was successful; on exploration, an ileal-ileal form had been found. She was admitted to the pediatric intensive care unit (PICU) one day postoperatively, and her altered mental status resolved in the immediate postoperative period and well-formed stools.

## 3. Discussion

The incidence of acute intussusception is estimated to be between 1% and 8% depending on the series [1–3]. The ileal-ileal form is more frequent than the jejunojejunal form [4].

The classic triad of signs includes paroxysmal abdominal pain, vomiting, and bloody stools. It is rarely complete at present in the industrialized countries.

Among acute intussusception cases, 90% are idiopathic, with no obvious trigger [5]. Its peak of incidence is correlated with the seasonality of viral gastroenteritis, and 30% of cases are preceded by a viral infection, whether it is an infection of the upper respiratory tract, acute otitis media, flu-like syndrome, or gastroenteritis. Whether viral or bacterial, enteritis is a powerful predictor of acute intussusception [6].

Pathophysiology of altered mental status is not yet clear [7]. Conway shares the opinion of action on a nervous system that is particularly receptive to various neuromediators (endotoxins, intestinal neurohormones, endorphins, etc.) released by the ischemic bowel [8]. Also, rapidly developing dehydration and shock can contribute to lethargy in the pediatric population [9].

In our case, there was no evidence of shock, but the infant was moderately dehydrated.

Endogenous opioid poisoning is hypothesized to be the cause of the miosis and may hint at the diagnosis and aid in early management [7], but miosis is not always present, and the effectiveness of naloxone is very inconsistent and in any case difficult to interpret in the context of fluctuating consciousness disorders [10].

Pumberger et al. [11] described 13 cases of intussusception where consciousness disorders preceded gastrointestinal symptoms. On the contrary, the actual frequency of neurologic symptomatology in children with all is unknown.

However, the neurological deterioration can be associated with digestive symptoms or isolated, totally dominating the clinical presentation, since it was referred in 1.7% of cases of the Kleizen series [12].

So, when the neurological signs are predominant or the only dysfunction, the clinician is forced to perform an extensive differential diagnosis in order to exclude other causes of acute encephalopathy: infectious, structural, vascular, hydroelectrolytic, toxic, endocrinological, autoimmune, metabolic, or epileptic [13]. In the present case report, the infant presented vomiting but the neurological signs were predominant, and even misleading: lethargy, hypotonia, and convulsions in a febrile context led us to achieve a more exhaustive infectious investigation, and the radiological assessment did not objectify a cause.

While most cases report required surgical reduction [7–9], in our case the patient underwent a surgical reduction with good outcome.

In addition, Maldonado and Takagishi recommend checking a stool for occult blood and an abdominal ultrasound in patients undiagnosed with altered mental status. This is particularly important in patients younger than 12 months [14].

## 4. Conclusion

The acute intussusception can be a differential diagnosis in infants and young children with neurological symptoms especially lethargy, and there are or not classic gastrointestinal signs. Early diagnosis in these cases reduces the time and cost of management while improving the prognosis.

## References

[1] Ein S. H., Stephens C. A. (1971). Intussusception: 354 cases in 10 years. *Journal of Pediatric Surgery*.

[2] Pollet J. E. (1980). Intussusception: a study of its surgical management. *British Journal of Surgery*.

[3] Kim Y. S., Rhu J. H. (1989). Intussusception in infancy and childhood: analysis of 385 cases. *International Surgery*.

[4] Pracros J. P., Louis D., Tran-Minh V. A., Deffrenne P., Morin de Finfe C. H. (1989). Invagination intestinale aiguë du nourrisson et de l’enfant. *EMC (Elsevier Masson SAS, Paris), Radiologie et imagerie médicale: Abdominale—Digestive*.

[5] Kennedy M., Liacouras C. A., Kliegman R. M., Stanton B. F., Schor N. F. (2011). Intussusception. *Nelson’s Textbook of Pediatrics*.

[6] Nylund C. M., Denson L. A., Noel J. M. (2010). Bacterial enteritis as a risk factor for childhood intussusception: a retrospective cohort study. *Journal of Pediatrics*.

[7] Dankoff S., Puligandla P., Beres A., Bhanji F. (2015). An unusual presentation of small bowel intussusception. *CJEM*.

[8] Conway E. E. (1993). Central nervous system findings and intussusception: how are they related?. *Pediatric Emergency Care*.

[9] Venkatesha G. A., Natarajan A. (2009). Neurological presentation of intussusception: case discussion and literature review. *Case Reports*.

[10] d’Escdenne M. M., Velin P., Filippigh P. (1996). Forme léthargique d’invagination intestinale aigui du nourrisson. *Archives de Pédiatrie*.

[11] Pumberger W., Dinhobl I., Dremsek P. (2004). Altered consciousness and lethargy from compromised intestinal blood flow in children. *American Journal of Emergency Medicine*.

[12] Kleizen K., Hunck A., Wijnen M., Draaisma J. (2009). Neurological symptoms in children with intussusception. *Acta Paediatrica*.

[13] Domínguez-Carral J., Puertas-Martín V., Carreras-Sáez I., Maraña-Pérez A. L., Escobar-Delgado T., García-Peñas J. J. (2014). Manifestaciones neurológicas de la invaginación intestinal. *Anales de Pediatría*.

[14] Maldonado L., Takagishi J. (2014). Four-month-old infant with intussusception presenting as altered mental status. *SAGE Open Medical Case Reports*.

